# Functional analysis of the mammalian RNA ligase for IRE1 in the unfolded protein response

**DOI:** 10.1042/BSR20160574

**Published:** 2017-03-02

**Authors:** Juthakorn Poothong, Witoon Tirasophon, Randal J. Kaufman

**Affiliations:** 1 Institute of Molecular Biosciences, Mahidol University, Salaya, Nakhon Pathom 73170, Thailand; 2 Degenerative Diseases Program, Sanford Burnham Prebys Medical Discovery Institute, 10901 N. Torrey Pines Rd., La Jolla, CA 92037, U.S.A.

**Keywords:** archease, human RtcB, human IRE1α/XBP1, Rlg1p, rlg1-100

## Abstract

The unfolded protein response (UPR) is a conserved signalling pathway activated on the accumulation of unfolded proteins within the endoplasmic reticulum (ER), termed ER stress. Upon ER stress, *HAC1*/*XBP1* undergoes exon/intron-specific excision by inositol requiring enzyme 1 (IRE1) to remove an intron and liberate the 5′ and 3′ exons. In yeast, the 5′ and 3′ *HAC1* exons are subsequently ligated by tRNA ligase (Rlg1p), whereas *XBP1* ligation in mammalian cells is catalysed by a recently identified ligase, RtcB. In the present study, RNA ligase activity of the human RtcB (hRtcB) involved in the unconventional splicing of *XBP1*/*HAC1* mRNA was explored in an *rlg1-100* mutant yeast strain. Distinct from *Escherichia coli* RtcB and Rlg1p, expression of hRtcB alone inefficiently complemented *HAC1*/*XBP1* splicing and the hRtcB cofactor (archease) was required to promote enzymatic activity of hRtcB to catalyse RNA ligation.

## Introduction

In eukaryotic cells, one-third of total proteins are destined to the secretory pathway, intracellular compartments, the plasma membrane or the extracellular space [1]. Perturbation of endoplasmic reticulum (ER) protein-folding homoeostasis results in the accumulation of unfolded proteins in the ER lumen, termed ‘ER stress’, which can be lethal to the cell. To restore protein-folding homoeostasis, the unfolded protein response (UPR) in the ER lumen is activated by the conserved sensor inositol requiring enzyme 1 (IRE1) as well as two additional UPR sensors in metazoan cells, activating transcription factor 6 (ATF6) and protein kinase RNA (PKR)-like ER kinase (PERK) [2,3]. Under ER stress, IRE1 forms oligomers to promote trans-autophosphorylation resulting in the allosteric activation of its RNase activity [4].

**Figure 1:**
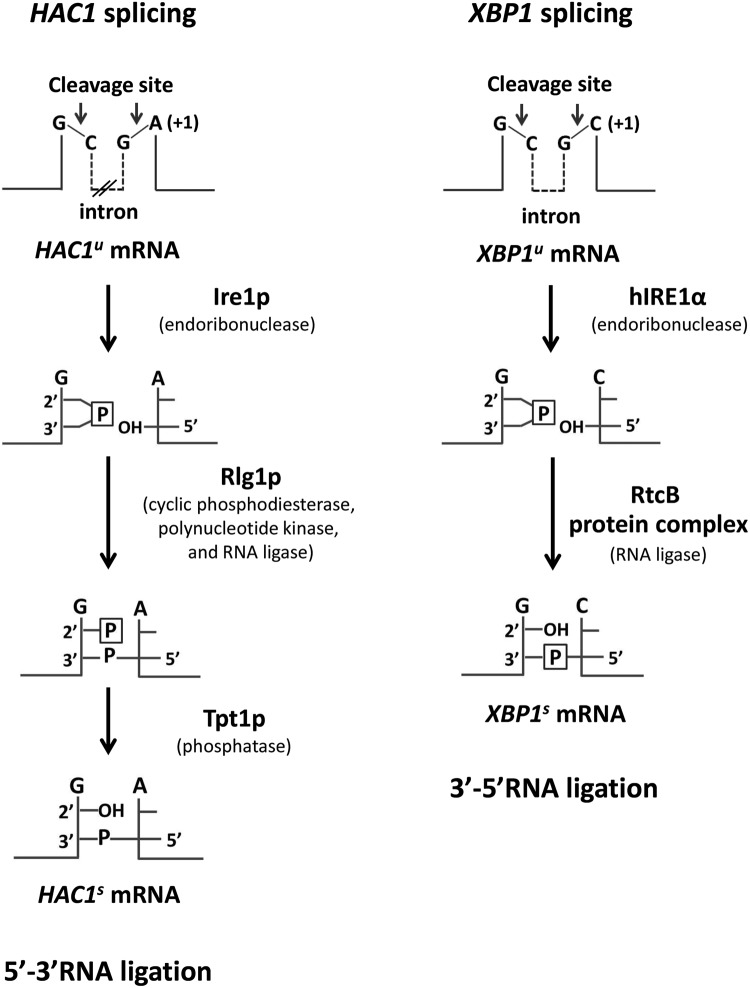
*HAC1* and *XBP1* splicing *HAC1*/*XBP1* are cleaved specifically by Ire1 (Ire1p/hIRE1α) endo-RNase to generate a 2′,3′-cyclic phosphate and 5′-OH end at 3′ and 5′ exons respectively.  In yeast, the two ends are modified before ligation by a multifunctional protein Rlg1p with cyclic phosphodiesterase, polynucleotide kinase and RNA ligase activities leaving a 2′-phosphate at the splice junction. The 2′-phosphate is finally removed by Tpt1p phosphatase. This ligation is defined as 5′–3′ RNA ligation.  In mammals, the two ends are directly ligated by the RtcB protein complex activity via 3′–5′ RNA ligation.  This protein complex is composed of five subunits: ASW, CGI-99, DDX1, FAM98B and archease. The nucleotides at +1 position in the 3′-splice site junction of *HAC1* and *XBP1* are indicated. P represents the phosphate group derived from 2′,3′-cyclic phosphate.

In *Saccharomyces cerevisiae*, the unspliced HAC1 (*HAC1^u^*) mRNA precursor is recognized and cleaved by active Ire1p to remove a 252-nt intron [5,6]. In a similar manner, unspliced *XBP1* (*XBP1^u^*) undergoes human IRE1α (hIRE1α)-mediated cleavage to remove a 26-nt intron in human cell. As a consequence of Ire1p and hIRE1α cleavage, a 2′,3′-cyclic phosphate at the 3′-end of the 5′ exon and a 5′-OH at the 5′-end of the 3′ exon are generated (Figure 1). Following the cleavage reaction in *HAC1* splicing, Rlg1p conducts its enzymatic activities (cyclic phosphodiesterase, polynucleotide kinase and RNA ligase) to catalyse RNA ligation between the two exons via 5′–3′ RNA ligation generating spliced *HAC1* (*HAC1^s^*) transcript. By this mechanism, the phosphate utilized for phosphodiester bond formation between two exons is obtained from a nucleotide triphosphate cofactor, whereas the unincorporated 2′ phosphate that originates from the 2′,3′-cyclic phosphate precursor at the splice-site junction is ultimately removed by Tpt1 phosphatase [7,8]. In contrast, RNA ligation in *XBP1* splicing mediated by RtcB goes through a 3′–5′ RNA ligation in which the phosphodiester bond is directly formed between the phosphate derived from the 2′,3′-cyclic phosphate precursor and the 5′-OH generating spliced *XBP1* (*XBP1^s^*) [9,10] (Figure 1).

RtcB, the catalytic subunit of the tRNA ligase complex and its cofactor (archease) have been identified as the RNA ligase that mediates tRNA splicing [9–11] and *XBP1* mRNA splicing both *in vitro* and *in vivo* in many organisms [12–14], except in fungi and plants as they have Rlg1p and *Arabidopsis* *thaliana* tRNA ligase respectively, for mediating tRNA splicing [15,16]. Archease is required for accelerating RtcB RNA ligation with GTP and is Mn^2+^-dependent [17]. However, some RtcBs are archease-independent in mediating RNA ligation such as *Thermus thermophilus* RtcB [18].

Interestingly, *Escherichia coli* RtcB is able to compensate for Rlg1p function in *HAC1* splicing in *rlg1*-defective yeast [10]. Likewise, *E. coli* RtcB with a possible accessory role of mammalian RtcB in *HAC1*/*XBP1* splicing is not well characterized. In the present study, we used an *rlg1-100* yeast strain as a surrogate platform for elucidating whether human RtcB (hRtcB) could mediate RNA ligation to provide insight into the *HAC1*/*XBP1* splicing event. We demonstrate that hRtcB is unable to catalyse RNA ligation by itself unless human archease is simultaneously expressed.

## Materials and methods

### Yeast strains and bacterial strains

*S. cerevisiae* Δ*ire1*/Δ*hac1* and the Δ*ire1*/Δ*hac1*/*rlg1-100* triple deletion yeast strain was developed from CF203 [16]. The *HAC1* and *URA3* gene loci of CF203 were replaced with *Zeocin^r^* and *kanMX* respectively, by double homologous recombination. *E. coli* DH5α was used for propagation and construction of all plasmids.

### Plasmids construction

pYES-Ire1 and pYES-hIRE1α were used for expressing exogenous yeast Ire1 and human IRE1α in yeast cells respectively. These two expression plasmids were constructed in the pYES2 vector under *GAL1* inducible promoter as previously described [19].

YCplac111-mtHAC1 was constructed for expressing *HAC1^u^* mRNA that carries a single point mutation (adenine to cytosine) at the +1 position in the 3′-splice site junction. We have recently demonstrated that wild-type *HAC1* was unable to be spliced by hIRE1α unless adenine (A) at +1 position in the 3′-splice site junction was substituted with cytosine (C) [20]. To construct this expression plasmid, the 2.5-kb *mtHAC1* coding sequence including the *ADH1* promoter and *CYC1* transcription terminator from pTB-mtHAC1 was subcloned into YCplac111 between the SmaI and XbaI sites. YCplac111-dmXBP1-3′BE was constructed as an *dmXBP1^u^* expression plasmid in yeast. The 3′BE refers to the 3′ bipartite element in the 3′-UTR of *HAC1* that acts as an ER-membrane targeting signal [21]. We utilized YCplac111-mtHAC1 as a template to replace the *mtHAC1* coding sequence with the unspliced *Drosophila melanogaster XBP1* (*dmXBP1^u^*) coding sequence from pcDNA-dmXBP1^u^ [22].

pTB-hRtcB was created to express hRtcB. The 1645-bp fragment from *hRtcB* cDNA was amplified from HeLa cells using HSPC117F and HSPC117R primers and cloned into pTB326 between the EcoRI and KpnI restriction sites. pTB-RtcB was created as an expression plasmid for *E. coli* RtcB in yeast. The bacterial RtcB coding sequence was amplified from DH5α genomic DNA using RtcB primers (Table 1). The 1300-bp PCR product corresponding to *E. coli RtcB* coding sequence was inserted into pTB326 between the KpnI and EcoRI sites.

**Table 1:** Primers used in the present study

Primers	Sequence (5′-3′)
HSPC117 F	TGA ATT CAG ATC TGT GCT CTG AGA AGC CGG ACT AC
	EcoRl
HSPC117 R	GAG GTA CCC AGT CCA CTT CCA CTT CAG AG
	Kpnl
RtcB F	GGG TAC CTT ATC ATC GAC AGG CTC AGC
	Kpnl
RtcB R	GGA ATT CTC TCT AGA CAC CAT GAA TTA CGA ATT ACT G
	EcoRl
ARCH F	GCG GAT CCT AGA GCG GAA GTA GTA ACT C
	BamHl
ARCH R	CCA GTC GAC ACA GTT CTT CGT AGG AGT C
	Sall
HAC1flkF	GCC CAA GAG TAT GCG CGA TTC CG
HAC1flkR	ACC CTC TTG CGA TTG TCT TCA TG
HAC 1^s^ F	GCC CAA GAG TAT GCG CGA TTC CG
HAC 1^s^ R	CAA ACC TGA CTG CGC TGC TGG
hlRE1α F	TC TAT CCA TGC CCA ATG CAC ACG
hlRE1αR	GTC GCT CAC GTC CTG GAA GAA C
lre1 F	ACC GCA TCC CTT TAA TCC TGG TGA
lre1 R	GAC TTC CAT CGT TCA CAG CAC CTT C
dmXBP1^s^F	CGA ACT GAA GCA GCA ACA GCA G
dmXBP1^s^F2	GGC TGG ATC CCA GCC CAA GGC CAA GAA GC
dmXBP1^s^R	GTA TAC CCT GCG GCA GAT CCA AGG
actin F	CAT CTA TCG TCG GTA GAC CAA G
actin R	GGA GCA ATG ATC TTG ACC TTC ATG
qRT KAR2F	TGG GTG GTG GTA CTT TCG ATG TCT
qRT KAR2R	AGC TAG GGC CTT GTT GTT GTC AGA
qRT actin F	CAC GTC GTT CCA ATT TAC GCT GGT
qRT actin R	TCG AAG TCC AAG GCG ACG TAA CAT

pAG-archease was created as a recombinant plasmid for expression of human archease in yeast cells. The cDNA fragment of human archease was produced by PCR using ARCH primers and inserted into pTEF413 between the BamHI and SalI sites. This vector is an intermediate, for which the human archease including *TEF* promoter and *CYC1* terminator from pTEF413 backbone was further blunt-end ligated with pAG26. All PCR reactions were performed using Phusion DNA polymerase (Thermo Scientific) and sequences were confirmed by DNA sequencing. Primers used for constructing each recombinant plasmid are shown in Table 1.

### Exogenous gene expression in yeast

The Δ*ire1*/Δ*hac1* or Δ*ire1*/Δ*hac1*/*rlg1-100* triple deleted yeast strain was transformed with recombinant plasmid for expressing *IRE1* (pYES-Ire1 or pYES-hIRE1α), unconventional RNA substrate (YCplac111-mtHAC1 or YCplac111-dmXBP1-3′BE), RNA ligase (pTB-hRtcB or pTB-RtcB) and pAG-archease using an electroporation protocol [23]. The transformants were selected on a synthetic dropout medium that corresponded to auxotrophic marker of each vector backbone containing 2% (v/v) D-glucose. To determine the RNA ligase activity of candidate proteins, yeast cells were cultured in dropout medium containing 2% (v/v) D-glucose. The *IRE1* expression was induced by addition of 2% (v/v) D-galactose. Finally, cells were exposed to ER stress by treatment with dithiothreitol (DTT). For maintaining the pAG expression vector, yeast cells were cultured in medium supplemented with hygromycin B (250 μg/ml). A similar protocol was used for testing the ability for Rlg1p to complement hIRE1α/*XBP1* splicing in the Δ*ire1*/Δ*hac1* double deleted yeast strain.

### RT-PCR

Yeast cells were pretreated with lyticase (Sigma) in buffer containing 1 M sorbitol, 0.1 M EDTA, pH=8.0 and 0.1% (v/v) β-mercaptoethanol. The reaction was incubated at 30ºC for 30 min and then the yeast-cell pellet was resuspended with 1 ml TRIzol Reagent (MRC) and RNA isolation was performed following the manufacturer’s instructions. First-strand cDNA was synthesized from DNase I-treated RNA using oligo d(T) with Improm-II reverse transcriptase (Promega). The *HAC1^u^* and *HAC1^s^* transcripts were monitored by RT-PCR using primers that flank the 252-nt intron (HAC1flkF and HAC1flkR). Alternatively, the *HAC1^s^* transcript was selectively monitored using HAC1^s^F and HAC1^s^R primers. In a similar manner, *dmXBP1^s^* transcripts were measured using dmXBP1^s^F and dmXBP1^s^R primers. The transcription levels of *hIRE1*α, *Ire1*, *hRtcB*, *E. coli RtcB*, *archease* and *actin* were measured using specific primers (Table 1). The *HAC1^s^* and *dmXBP1^s^* PCR detections were performed at 69 and 74.7ºC annealing temperatures respectively. The PCR was performed using DNA polymerase (Thermo Scientific).

### Quantitative PCR (qPCR)

The same preparations of cDNAs used in RT-PCR were diluted 10-fold before analysis of gene expression by qRT-PCR using the SYBR fluorescent dye (KAPA). The *HAC1^s^*, *dmXBP1*^s^, *KAR2* and *actin* mRNAs were monitored with specific primers (Table 1). The PCR protocol for analysing *HAC1^s^* was performed on following parameters: 95ºC for 3 min followed by 40 cycles of 95ºC for 10 s, 69ºC for 30 s and 72ºC for 20 s. The quantification of *dmXBP1^s^*, *KAR2* and *actin* mRNAs was performed using a similar protocol except the annealing temperatures being 74.7ºC, 57ºC and 57ºC respectively. The amplification data were analysed according to 2^−^^ΔΔ*C*^_T_ method normalizing with *actin* mRNA levels.

### Western blot of hRtcB

To determine expression level of hRtcB, yeast cells were cultured, pelleted and resuspended in 4× lysis buffer [24]. Cell suspensions were then boiled for 5 min. The yeast cell extract was resolved on 4–15% gradient gels (Bio–Rad Laboratories). The blot was probed with rabbit polyclonal antibody hRtcB protein (Santa Cruz Biotechnology) and then the antigen–antibody complexes were monitored by horseradish-peroxidase-conjugated with anti-rabbit IgG (Sigma–Aldrich) using the West Dura chemiluminescent substrate (Thermo Scientific) detection system.

## Results

### Rlg1p inefficiently rescues hIRE1α-mediated splicing in yeast cells

**Figure 2:**
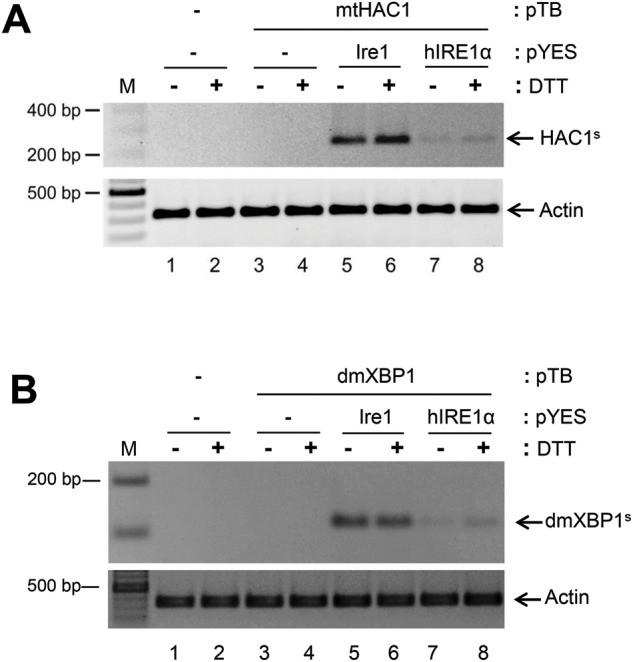
Rlg1p is able to mediate *XBP1* splicing in yeast The Δ*ire1*/Δ*hac1*/*RLG1* yeast strain was transformed with a recombinant plasmid to express *mtHAC1*/*dmXBP1* with *hIRE1α* or *Ire1*. Splicing of *dmXBP1*/*mtHAC1* was analysed after induction of *IRE1* expression for 17 h with D-galactose and subsequently treating the cells with 5 mM DTT for 2 h. Splicing was quantified by RT-PCR using splice-specific primers. (**A**) RT-PCR of *HAC1^s^*. (**B**) RT-PCR of spliced *dmXBP1* (*dmXBP1^s^*). *Actin* transcription level was included as internal control. M: GeneRuler DNA ladder.

Previous studies demonstrated that Rlg1p is essential for splicing of Ire1p/*HAC1* mRNA in yeast cells [16]. Interestingly, we found that Rlg1p is also able to complement hIRE1α/mt*HAC1* splicing in ∆*ire1*/∆*hac1*/*RLG1* [20], however, *HAC1* splicing efficiency was much less than Ire1p-mediated splicing (Figure 2A, compare lanes 7–8 with 5–6 and Supplementary Figure S1A). Since *HAC1* mRNA is not a natural substrate for hIRE1α, it is possible that the *HAC1* splicing substrates generated by Ire1p and hIRE1α are different. To test this hypothesis, we analysed *XBP1* mRNA as a splicing substrate to investigate hIRE1α cleavage activity. Using the same analysis platform, *dmXBP1^u^* was co-expressed with *hIRE1α*. Under ER stress conditions induced by DTT, a single PCR product of 106 bp corresponding to *dmXBP1^s^* was clearly detected exclusively upon *hIRE1α* co-expression, but not presented upon *dmXBP1* expression alone (Figure 2B, compare lanes 5–8 with 3–4, Supplementary Figure S1B). By Western blot analysis, the predicted pXBP1^u^ (~37 kDa) and pXBP1^s^ (~50 kDa) were undetectable from yeast cell lysates (results not shown). This might be due to instability of XBP1 protein in the yeast cells [25]. Notably, overexpression of *hIRE1α* was capable to mediate *dmXBP1* splicing even under non-stress condition as we previously reported [19]. Interestingly, we found that *dmXBP1* could be processed by Ire1p similar to hIRE1α mediated splicing. The *dmXBP1^s^* was easily detected upon *Ire1* co-expression (Figure 2B, lanes 5 and 6). However, *dmXBP1* substitution did not increase the hIRE1α splicing efficiency in this wild-type tRNA ligase *RLG1* yeast strain. Regardless of the mRNA substrate, our results suggest that Rlg1p is not compatible to complement the hIRE1α-mediated splicing process.

### *E. coli* RtcB, not hRtcB alone can complement HAC1/XBP1 splicing

**Figure 3:**
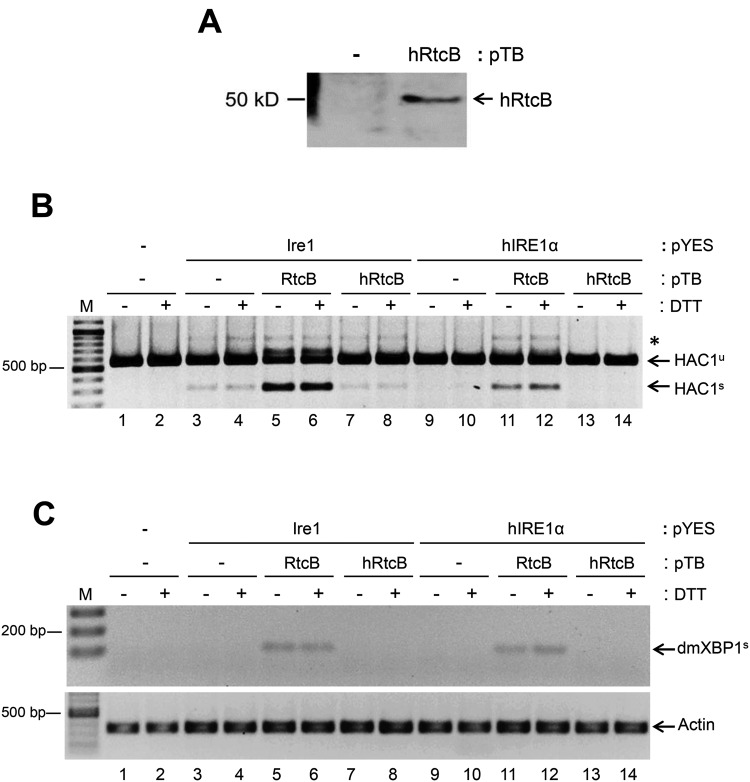
*E. coli* RtcB efficiently mediate *HAC1*/*XBP1* splicing in yeast The Δ*ire1*/Δ*hac1*/*rlg1-100* yeast strain was transformed with three different expression plasmids to express *mtHAC1*/*dmXBP1*, *hIRE1α*/*Ire1* and *hRtcB*/*E.coli RtcB* (*RtcB*). The splicing efficiencies of *dmXBP1* and *mtHAC1* were measured after induction of *IRE1* expression with D-galactose for 17 h and ER stress by treatment with 5 mM DTT for 2 h. (**A**) Western blot analysis of hRtcB. (**B**) RT-PCR of *HAC1* using an intron-flanking primers. Asterisk (*) represents a hybrid between unspliced and spliced forms of mutant *HAC1* transcript. (**C**) RT-PCR of *dmXBP1^s^* using specific primers. *Actin* mRNA level was used as an internal control. M: GeneRuler DNA ladder.

It was reported that *rlg1*-*100* deficiency severely impairs *HAC1* splicing but does not affect the pre-tRNA maturation process [16]. Taking this advantage, we asked whether hRtcB can compensate for Rlg1p to complement the hIRE1α requirement in *HAC1* mRNA splicing. For this purpose, we transformed Δ*ire1*/Δ*hac1*/*rlg1*-*100* yeast strain with three different recombinant plasmids encoding *mtHAC1*, hIRE1α or Ire1p and hRtcB. From the transformed yeast-cell lysates, the hRtcB protein (~55 kDa) was detected as predicted (Figure 3A). We found that *rlg1*-*100* was not totally defective in RNA ligase activity. Indeed, it is still sufficient to complement *HAC1* splicing i.e. there is a faint band of *HAC1^s^* appearing in *Ire1* co-expressing cells (Figure 3B, lanes 3 and 4). Although, *HAC1* splicing was markedly rescued when *E. coli RtcB* was co-expressed with *Ire1* included as positive control (Figure 3B, lanes 5 and 6). Unexpectedly, *HAC1* splicing process was defective in yeast with co-expression of *hRtcB* (Figure 3B, lanes 7 and 8). Even though a faint band intensity of *HAC1^s^* was detected upon *hRtcB* co-expression, it was not significantly different compared with *Ire1* expression alone (Supplementary Figure S2A). A similar result was obtained in the presence of *hIRE1α* co-expression (Figure 3B, lanes 9–14).

To confirm our finding, the RNA ligase activity of hRtcB was further tested by its ability to ligate the *dmXBP1* RNA substrate. Surprisingly, *rlg1*-*100* did not mediate the ligation reaction (Figure 3C, lanes 3–4 and 9–10) in contrast with *HAC1* splicing. As previous observed, the *dmXBP1* splicing was exclusively complemented only by *E. coli RtcB* co-expression (Figure 3C, lanes 5–6 and 11–12) but not in *hRtcB* co-expression (Figure 3C, lanes 7–8 and 13–14, Supplementary Figure S2B). We propose that hRtcB by itself is not able to ligate RNA and the reaction required an additional cofactor(s) for hRtcB RNA ligase-mediated mRNA splicing.

### Archease facilitates hRtcB RNA ligase activity

**Figure 4:**
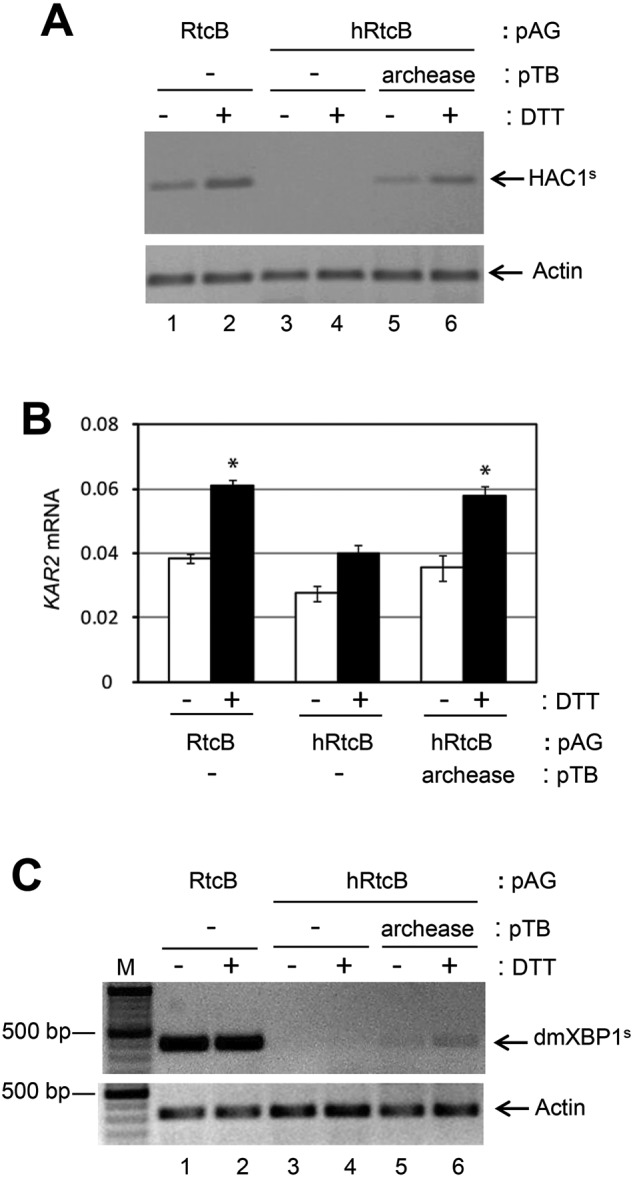
Archease is required for hRtcB RNA ligase activity The Δ*ire1*/Δ*hac1*/*rlg1-100* yeast strain was transformed with four different plasmids to express *hIRE1α*/*Ire1*, *mtHAC1*/*dmXBP1*, *hRtcB*/*RtcB* and *archease*. The effect of archease in hRtcB function was determined. (**A**) RT-PCR of *HAC1^s^* using *HAC1^s^*-specific primers after induction of *IRE1* expression for 18 h including 2-h DTT treatment. (**B**) qPCR of *KAR2*. The mRNA level was normalized with yeast *actin* mRNA level and is shown as mean ± S.E.M. and subjected to one-way ANOVA test (*P<0.01, compared with hIRE1α, *mtHAC* and hRtcB co-expression at the same condition). (**C**) RT-PCR of *dmXBP1^s^* using splice-specific primers (dmXBP1^s^F2 and dmXBP1^s^R) (Table 1) after induction of *hIRE1α* expression for 45 h, then a 3-h DTT treatment. *Actin* mRNA levels were used as an internal control. M: GeneRuler DNA ladder.

Distinct from Rlg1p, mammalian RtcB is associated with ASW, DDX1, FAM98B, CGI-99 and archease in the tRNA ligase complex [9,26 ]. Of all these associated proteins, archease is known as an activator for RtcB to mediate *in vitro* RNA ligation of cleaved pre-tRNA transcripts [27]. However, the ability of human archease to directly stimulate hRtcB in the *HAC1*/*XBP1* splicing reaction *in vivo* has not been reported. Hence, the hRtcB RNA ligase activity was further analysed upon *archease* co-expression. We found that *HAC1* splicing in yeast was still defective upon *archease* expression alone. This indicates that archease has no RNA ligase activity and the *rlg1-100* cannot mediate *HAC1* splicing under these conditions. Consistently, *HAC1* splicing was complemented with *E. coli RtcB* (Figure 4A, lanes 1 and 2) but not with *hRtcB* expression alone (Figure 4A, lanes 3 and 4). Interestingly, *HAC1* splicing was restored when *archease* was simultaneously expressed with *hRtcB* (Figure 4A, lanes 5 and 6, Supplementary Figure S3A). We observed the expression of *KAR2*, a downstream target of the Hac1 transcription factor, was increased upon *archease* co-expression (Figure 4B). The collaboration between hRtcB and archease in mediating RNA ligation was confirmed upon analysis of *dmXBP1* splicing (Figure 4C and Supplementary Figure S3B). Taken together, our results indicate that hRtcB and archease are fundamental subunits required for RNA ligase activity in the unconventional splicing reaction.

## Discussion

In the present study, we reconstituted the entire splicing process of *XBP1*/*HAC1* in the yeast mutant strain *rlg1-100*. Using this system, we studied functional roles of hRtcB in *HAC1*/*XBP1* splicing. Our results demonstrate that the hRtcB subunit expressed in yeast cell cannot complement *HAC1*/*XBP1* splicing as previous attempts to identify the RNA ligase using a purified fly RtcB and hRtcB from HEK293T cells as candidate protein, both were unable to complement *in vitro* splicing of *XBP1*/hIRE1α [28]. In the present study, we demonstrated that hRtcB RNA ligase activity is recruited when its cofactor (archease) is co-expressed in yeast cells. The exact functional role of archease to promote hRtcB RNA ligase activity in *XBP1* splicing is unclear. It likely involves a guanylylation process that is an initial step to activate the archaeal RtcB RNA ligase activity [17]. Although, the hRtcB and archease function involving RNA ligation was previously reported by RNAi knockdown where *XBP1* splicing was almost completely impaired when *hRtcB* and *archease* were simultaneously depleted, whereas knockdown of either *hRtcB* or *archease* alone did not effectively diminish splicing [10]. However, by RNAi-mediated RtcB knockdown, the RtcB-associated protein *DDX1* and *FAM98B* were unexpectedly co-depleted with *RtcB* consistent with *RtcB* conditional knockout mice [9,12].

Supporting our findings, RtcB is the responsible for RNA ligase in pre-tRNA splicing that occurs in the nucleus, however, some RtcBs are found at the ER membrane where *XBP1^u^* mRNA and hIRE1α are located [12,26]. RtcB is thought to interact with *XBP1^u^* mRNA specifically at intron-flanking regions [29]. However, our data not only demonstrated that hRtcB can catalyse *dmXBP1* ligation but it can also mediate *HAC1* ligation, although the putative RtcB binding site in *XBP1^u^* is not present in *HAC1^u^* mRNA. Likewise, Rlg1p and *E. coli* RtcB can ligate both *dmXBP1* and *HAC1* mRNA. Together the findings suggest that RNA ligase and RNA substrate interactions are not sequence specific, for example, it was shown that RtcB associates with hIRE1α at the ER membrane [12]. Thus, the RNA ligase may indirectly interact with its RNA substrate through IRE1. By this scenario, it is implicated that Rlg1p and other RtcBs associate with hIREα as hRtcB do during *XBP1* splicing in yeast.

In the present study, we showed that hRtcB RNA ligase activity on *XBP1*/*HAC1* splicing is very low compared with *E. coli* RtcB activity (Supplementary Figures S3A and S3B). An *in vitro*-ligation assay suggested that DDX1 helicase is another factor required for RtcB activation to mediate pre-tRNA ligation [27]. Therefore, it is possible that DDX1 is required for full activation of hRtcB in *XBP1* splicing in addition to archease. This is in contrast with *E. coli* RtcB that is able to catalyse RNA ligation by itself without any cofactor [10]. Besides DDX1, it is interesting to investigate the exact function of other RtcB cofactors (ASW, FAM98B and CGI-99) in the RNA ligase complex. This might be useful for discriminating functional roles of each component of the tRNA ligase complex in pre-tRNA maturation process, *XBP1* splicing and possibly RIDD (regulated IRE1-dependent decay) [30]. The IRE1α cleavage reaction generates a 5′-OH and a 2′,3′-cyclicphosphate terminus that are potential substrates for RtcB.

## Abbreviations

dmXBP1^s^: spliced* Drosophila melanogaster *XBP1
dmXBP1^u^: unspliced* Drosophila melanogaster *XBP1
ER: endoplasmic reticulum
HAC1^s^: spliced HAC1
HAC1^u^: unspliced HAC1
hIRE1α: human IRE1α
hRtcB: human RtcB
IRE1: inositol requiring enzyme 1
qPCR: quantitative PCR
UPR: unfolded protein response
XBP1: X-box-binding protein 1
XBP1^s^: spliced XBP1
XBP1^u^: unspliced XBP1
3′BE: 3′ bipartite element
